# Prognosis of Spontaneous Pneumothorax/Pneumomediastinum in Coronavirus Disease 2019: The CoBiF Score

**DOI:** 10.3390/jcm11237132

**Published:** 2022-11-30

**Authors:** Wongi Woo, Vincent Kipkorir, Adina Maria Marza, Shadi Hamouri, Omar Albawaih, Arkadeep Dhali, Wooshik Kim, Zarir F. Udwadia, Abdulqadir J. Nashwan, Nissar Shaikh, Alessandro Belletti, Giovanni Landoni, Diego Palumbo, Sarya Swed, Bisher Sawaf, Danilo Buonsenso, Inês Pimenta, Filipe André Gonzalez, Giuseppe Fiorentino, Muhammad Redzwan S. Rashid Ali, Alvaro Quincho-Lopez, Mohammad Javanbakht, Ayat Alhakeem, Muhammad Mohsin Khan, Sangam Shah, Moezedin Javad Rafiee, Sri Rama Ananta Nagabhushanam Padala, Sebastian Diebel, Seung Hwan Song, Du-young Kang, Duk Hwan Moon, Hye Sun Lee, Juyeon Yang, Luke Flower, Dong Keon Yon, Seung Won Lee, Jae Il Shin, Sungsoo Lee, International COVID-19 Pneumothorax Working Group (ICP-WG)

**Affiliations:** 1 Department of Thoracic and Cardiovascular Surgery, Gangnam Severance Hospital, Yonsei University College of Medicine, Seoul 03722, Republic of Korea; 2 Department of Human Anatomy, School of Medicine, University of Nairobi, Nairobi 00100, Kenya; 3 Department of Surgery, “Victor Babes” University of Medicine and Pharmacy, 300041 Timisoara, Romania; 4 Department of General Surgery & Urology, King Abdullah University Hospital, Jordan University of Science & Technology, Irbid 22110, Jordan; 5 Department of General Surgery and Special Surgery, Faculty of Medicine, Al- Balqa’ Applied University, Al-Salt 19117, Jordan; 6 Institute of Postgraduate Medical Education and Research, Kolkata 700020, India; 7 Department of Thoracic and Cardiovascular Surgery, National Medical Center, Seoul 04564, Republic of Korea; 8 Breach Candy Hospital and Research Center, Mumbai 400026, India; 9 Critical Care Department, Hazm Mebaireek General Hospital (HMGH), Hamad Medical Corporation (HMC), Doha 576214, Qatar; 10 Surgical Intensive Care Department, Hamad General Hospital (HGH), Hamad Medical Corporation (HMC), Doha 576214, Qatar; 11 Department of Anesthesia and Intensive Care, IRCCS San Raffaele Scientific Institute, 20132 Milan, Italy; 12 Department of Radiology, IRCCS San Raffaele Scientific Institute, 20132 Milan, Italy; 13 Faculty of Medicine, Aleppo University, Aleppo 15310, Syria; 14 Department of Internal Medicine, Hamad General Hospital, Doha 576214, Qatar; 15 Department of Woman and Child Health and Public Health, Fondazione Policlinico Universitario Agostino Gemelli, 00168 Rome, Italy; 16 Intensive Care Department, Hospital Garcia de Orta EPE, 2805-267 Almada, Portugal; 17 Sub-Intensive Care Unit and Respiratory Physiopathology Department, Cotugno-Monaldi Hospital, 80131 Naples, Italy; 18 Kpj Johor Specialist Hospital, Johor Bahru 80100, Malaysia; 19 Unidad de Investigación en Bibliometría, Universidad San Ignacio de Loyola, Lima 15024, Peru; 20 Nephrology and Urology Research Center, Baqiyatallah University of Medical Sciences, Tehran 1435916471, Iran; 21 Hamad Medical Corporation, Doha 576214, Qatar; 22 Maharajgunj Medical Campus, Institute of Medicine, Tribhuvan University, Kathmandu 44618, Nepal; 23 Babak Imaging Center, Tehran 1415943953, Iran; 24 McGill University Health Centre, Montreal, QC H4A 3J1, Canada; 25 Department of Anaesthesiology and Critical Care, All India Institute of Medical Sciences, Bhopal 462026, India; 26 Northern Ontario School of Medicine, Sudbury, ON P3E 2C6, Canada; 27 Department of Thoracic and Cardiovascular Surgery, Sanggye Paik Hospital, Inje University College of Medicine, Seoul 01757, Republic of Korea; 28 Department of Thoracic and Cardiovascular Surgery, Kangbuk Samsung Hospital, Sungkyunkwan University College of Medicine, Seoul 03063, Republic of Korea; 29 Biostatistics Collaboration Unit, Yonsei University College of Medicine, Seoul 03722, Republic of Korea; 30 William Harvey Research Institute, Barts and The London School of Medicine and Dentistry, Queen Mary University of London, London E1 4NS, UK; 31 Center for Digital Health, Medical Science Research Institute, Kyung Hee University Medical Center, Kyung Hee University College of Medicine, Seoul 02447, Republic of Korea; 32 Department of Precision Medicine, Sungkyunkwan University School of Medicine, Suwon 16419, Republic of Korea; 33 Department of Pediatrics, Yonsei University College of Medicine, Seoul 03722, Republic of Korea

**Keywords:** pneumothorax, pneumomediastinum, coronavirus disease, coronavirus disease 2019, prediction model

## Abstract

**Highlights:**

**What are the main findings?**

- Pneumothorax/pneumomediastinum developed without positive pressure ventilation among COVID-19 patients had high fatality.
- Presence of comorbidity, bilateral pneumothorax, and fever were related with in-hospital mortality among COVID-19 associated spontaneous pneumothorax/pneumomediastinum patients
- The CoBiF score (Co = comorbidity, Bi = bilateral pneumothorax, F = fever) well-predicted the early mortality of these patients.

**What is the implication of the main finding?**

- The CoBiF score was validated in multinational cohorts, and it could improve early recognition and treatment of COVID-19 pneumothorax.

**Abstract:**

**Objectives:** Pneumothorax and pneumomediastinum are associated with high mortality in invasively ventilated coronavirus disease 2019 (COVID-19) patients; however, the mortality rates among non-intubated patients remain unknown. We aimed to analyze the clinical features of COVID-19-associated pneumothorax/pneumomediastinum in non-intubated patients and identify risk factors for mortality. **Methods:** We searched PubMed Scopus and Embase from January 2020 to December 2021. We performed a pooled analysis of 151 patients with no invasive mechanical ventilation history from 17 case series and 87 case reports. Subsequently, we developed a novel scoring system to predict in-hospital mortality; the system was further validated in multinational cohorts from ten countries (*n* = 133). **Results:** Clinical scenarios included pneumothorax/pneumomediastinum at presentation (*n* = 68), pneumothorax/pneumomediastinum onset during hospitalization (*n* = 65), and pneumothorax/pneumomediastinum development after recent COVID-19 treatment (*n* = 18). Significant differences were not observed in clinical outcomes between patients with pneumomediastinum and pneumothorax (±pneumomediastinum). The overall mortality rate of pneumothorax/pneumomediastinum was 23.2%. Risk factor analysis revealed that comorbidities bilateral pneumothorax and fever at pneumothorax/pneumomediastinum presentation were predictors for mortality. In the new scoring system, i.e., the CoBiF system, the area under the curve which was used to assess the predictability of mortality was 0.887. External validation results were also promising (area under the curve: 0.709). **Conclusions:** The presence of comorbidity bilateral pneumothorax and fever on presentation are significantly associated with poor prognosis in COVID-19 patients with spontaneous pneumothorax/pneumomediastinum. The CoBiF score can predict mortality in clinical settings as well as simplify the identification and appropriate management of patients at high risk.

## 1. Introduction

During the coronavirus disease 2019 (COVID-19) pandemic, complications such as pneumothorax (PNx) and pneumomediastinum (PMEx) frequently occurred, both independently and more commonly in conjunction with each other [1]. PNx, which was observed previously in severe acute respiratory syndrome coronavirus infection and the Middle East respiratory syndrome, is significantly associated with poor prognosis [2,3]. PNx was also observed among patients with COVID-19, with an estimated incidence of 0.56–4.2%, which increased among patients requiring intensive care [1,4,5]. PNx and PMEx are adverse predictors of mortality, especially among critically ill patients with COVID-19 [6,7]. In particular, substantially high mortality (13.8–63.0%) from PNx/PMEx has been reported in patients with COVID-19, which is worse than that for PNx/PMEx arising from other respiratory etiologies [5,8]. 

Despite this correlation between PNx/PMEx and COVID-19, PNx/PMEx occurs after the implementation of invasive mechanical ventilation (IMV) in most cases, implying the possibility of adverse effects attributable to barotrauma or complications of critical care. Furthermore, due to its rarity, the existing literature on spontaneous PNx/PMEx in patients who did not receive IMV mainly includes case reports/series and small retrospective studies. Therefore, the true clinical features of COVID-19-associated spontaneous PNx/PMEx remain relatively unknown. Moreover, despite the high associated mortality, to our knowledge, no scoring system for in-hospital mortality in such patients exists.

Therefore, we aimed to comprehensively review spontaneous PNx/PMEx in COVID-19 patients by pooling individual patient data from previous case reports and case series and developing a novel scoring system to predict in-hospital mortality. Additionally, we evaluated multinational cohorts in collaboration with the International COVID-19 Pneumothorax Working Group (ICP-WG) and externally validated our new scoring system using this external dataset.

## 2. Patients and Methods

### 2.1. Search Strategy and Selection Criteria

This systematic review was registered with PROSPERO (CRD42022295621) and performed in accordance with the Preferred Reporting Items for Systematic Reviews and Meta-Analyses Protocols (PRISMA-P, Supplementary Table S1).

Articles on COVID-19 patients with PNx and/or PMEx who had not received IMV prior to PNx/PMEx onset, articles on patients with PNx/PMEx without a history of IMV who had been treated for COVID-19 within the past 3 months, and case reports and case series analyzing sufficient individual patient data were included. Articles reporting PNx/PMEx that developed after IMV or an unclear temporal relationship between the events and those on patients aged <14 years were excluded. Review articles, abstracts, letters to the editor, and articles that did not contain sufficient information on patient characteristics or outcomes were also excluded.

Two investigators (WW and VK) searched PubMed/Medline, Embase, and Scopus up to 21 December 2021. The search terms employed are described in detail in Supplementary Table S2. Discrepancies regarding the inclusion/exclusion of studies were discussed and resolved by consensus among four investigators (WW, VK, JIS, and SL). The initial search yielded 303 studies after the elimination of duplicates. After reviewing the abstracts and full texts of these articles, we identified 104 studies (87 case reports and 17 case series) that met the inclusion criteria (Supplementary Table S3). The PRISMA flow diagram of the selection process is depicted in Supplementary Figure S1.

We extracted data on patient demographics, clinical characteristics of PNx/PMEx, COVID-19-associated treatments, radiologic findings, treatments, and clinical outcomes from each eligible case report and case series. Since few studies reported the laboratory findings at presentation, which were not consistent between studies, they were not included in this analysis.

### 2.2. Data Collection

We recorded the first author, publication year, and country of origin for each eligible case report or case series and collected information on demographic and clinical characteristics, including sex, age, comorbidities, smoking history, COVID-19-specific medical treatments, symptoms, location of PNx/PMEx, existence of tension PNx or subcutaneous emphysema, chest computed tomography (CT) findings, treatments, duration of hospitalization, intensive care unit (ICU) admission, outcomes, and mortality. 

## 3. Statistical Analysis

Data on continuous variables (age, oxygen saturation, and duration of hospitalization) are presented as median and interquartile ranges after determining the normality of the distribution. Differences in these variables were compared using the Mann–Whitney U test. Fisher’s exact test was performed to compare categorical variables [9]. We performed logistic regression to determine the predictive factors for in-hospital mortality; logistic regression was considered suitable for analysis as all deaths occurred within 2 months of hospitalization. Variables with *p*-values < 0.05 in univariate analysis were entered into multivariate analysis, and the variables were selected by backward elimination with a two-tailed *p*-value of <0.05. The scoring system for in-hospital mortality was devised based on significant factors as follows: 

### 3.1. Step 1: Development and Internal Validation of the CoBiF Scoring System

Significant variables for in-hospital mortality (*p* < 0.05) in the multivariate logistic regression analysis were used to create the CoBiF scoring system. This scoring system was constructed using binary variables to ensure application ease. The score for each variable was measured based on its odds ratio (OR) and regression coefficient. Two variables (comorbidities and bilateral PNx) had similar magnitude for predicting the outcome (score for each: 1), whereas a third variable (fever at PNx/PMEx presentation) had a greater weight (score: 2). The summation of the three scores was used for the prediction model (score 0–4). If the value of any of these three parameters was missing, they were not included in the measurement of predictability. The discriminative power of the CoBiF model was assessed by plotting a receiver operating characteristic curve and calculating the area under the curve (AUC). Bias-corrected AUC was measured for internal validation, and the Mantel–Haenszel chi-square test was performed for calibration.

### 3.2. Step 2: External Validation

We developed an independent dataset with the same inclusion/exclusion criteria for external validation. First, authors who published retrospective studies in 2022 were approached, and their data were included [10,11,12,13]. Second, authors who reported COVID-19-related PNx/PMEx were contacted and encouraged to share data. This was performed by the ICP-WG. Finally, we gathered approved information on 133 patients from 10 countries. Thereafter, the performance of the CoBiF scoring model was assessed by computing the AUC.

All statistical analyses were performed using the Statistical Package for the Social Sciences (SPSS) for Windows version 25.0 (SPSS Inc., IBM Corporation, Armonk, NY, USA) and R version 4·0·4 (R Core Team, Vienna, Austria) and were supervised by a medical statistician (HSL).

## 4. Results

### 4.1. Demographics and Clinical Characteristics

The patient characteristics and specific COVID-19 treatments are outlined in Table 1. The study population included 151 patients, of whom >80% were men (128/151, 84.8%), and approximately 60% (58.2%, 88/151) were aged <60 years. In total, 54.4% (80/147) of patients had underlying medical conditions, such as hypertension (27.3%), diabetes (11.9%), obesity (11.9%), and other respiratory diseases (11.2%) (Table 1).

Most patients who developed PNx/PMEx commonly presented with dyspnea (83.8%), chest pain (44.7%), and fever (35.2%). PNx patients were diagnosed with radiologic features such as hyperlucency, hyperinflation, cavities, or cystic lesions. For PMEx-related radiologic findings, linear or curvilinear lucencies outlining mediastinal contours were mostly observed on CXR and/or CT. Other than these common features, chest CT revealed ground-glass opacity in most (81.5%) patients and emphysema (8.0%) and pleural effusion (8.1%) in some patients. Further, 37.0% of patients required intensive care, and the in-hospital mortality rate was high (23.2%).

### 4.2. Comparison between Pneumomediastinum and Pneumothorax ± Pneumomediastinum 

There were no differences in age, sex, comorbidity, clinical manifestations, COVID-19-associated treatments, and radiological findings between the PMEx and PNx ± PMEx groups, although obesity was more common in the PMEx group (Table 1). Due to disease-specific characteristics, most patients with PMEx were conservatively treated. However, more patients with PMEx than those with PNx received non-invasive ventilation. Overall, disease severity did not differ between the groups with respect to ICU admission, IMV requirement, and mortality.

### 4.3. Clinical Characteristics According to Clinical Scenarios

Supplementary Table S4 describes the patients’ clinical scenarios, classified into the following three categories: (1) group A, patients who presented to the hospital with PNx/PMEx (*n* = 68, 45.0%) and who had COVID-19 symptoms for a median of 7 days prior to the hospital visit; (2) group B, patients who developed PNx/PMEx during inpatient management for COVID-19 (*n* = 65, 43.1%) at a median of 10.0 days from admission; and (3) group C, patients who underwent re-admission within a median of 16.5 days due to development of PNx/PMEx after discharge following recent COVID-19 treatment (*n* = 18, 11.9%). There were no remarkable differences in the characteristics of patients who presented with the three clinical scenarios; however, the frequency of symptoms such as fever (*p* < 0.001), cough (*p* = 0.001), and mortality (*p* = 0.087) was higher in groups A and B than in group C.

### 4.4. Patient Characteristics According to Mortality

Overall, the in-hospital mortality rate was 23.2% (35/151). Table 2 demonstrates the differences between survivors and non-survivors. Compared to survivors, non-survivors were older (median 52.5 versus 67.0 years, *p* < 0.001) and had a frequency of comorbidities (*p* < 0.001), such as obesity (*p* = 0.007) and hypertension (*p* = 0.008). At presentation, the frequency of fever (*p* < 0.001), cough (*p* = 0.032), dyspnea (*p* = 0.026), and symptoms other than chest pain (*p* = 0.005) were higher in patients with adverse outcomes than in those with non-adverse outcomes. Non-survivors received more COVID-19-specific medical treatments, such as steroids (*p* = 0.004) and remdesivir (*p* = 0.037) than survivors. Although there were no differences in radiological findings, the frequency of bilateral PNx (*p* = 0.029) was higher among non-survivors than among survivors.

### 4.5. Risk Factor Analysis for in-Hospital Mortality

Multivariate logistic regression analysis revealed that the existence of comorbidities (OR: 3.87, 95% confidence interval [CI]: 1.27–11.9, *p* = 0.018), bilateral PNx (OR: 4.86; 95% CI: 1.08–21.9, *p* = 0.039), and fever at PNx/PMEx presentation (OR 24.1, 95% CI: 7.75–74.6, *p* < 0.001) were significantly associated with mortality (Table 3). The causes of death in these patients are enumerated in Supplementary Table S5. In total, 42.9% of patients died of respiratory failure due to the progression of COVID-19.

### 4.6. The CoBiF Scoring System for in-Hospital Mortality

**Figure 1:**
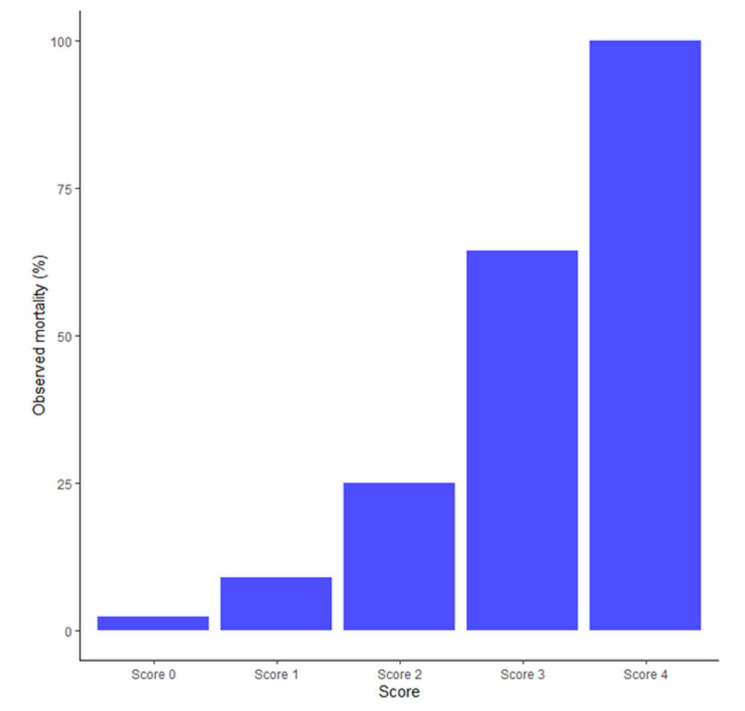
Probability of in-hospital mortality according to the CoBiF score.

A novel scoring system was designed to predict in-hospital mortality in patients who developed COVID-19-associated spontaneous PNx/PMEx. Multivariate logistic analyses revealed comorbidities, fever at PNx/PMEx presentation, and bilateral PNx as significant variables. Thus, the scoring system was named CoBiF (Co = comorbidities, Bi = bilateral PNx, F = fever) based on the components of this model. The predicted in-hospital mortality was proportional to the score—1.85%, 8.14%, 29.5%, 66.3%, and 90.3% with a CoBiF score of 0, 1, 2, 3, and 4, respectively (Figure 1). The AUC was 0.887 (95% CI: 0.822–0.951), and the Mantel–Haenszel chi-square test yielded a *p*-value of <0.0001, signifying a good fit between the model and observed data.

### 4.7. Internal and External Validation of the CoBiF Scoring System

**Figure 2:**
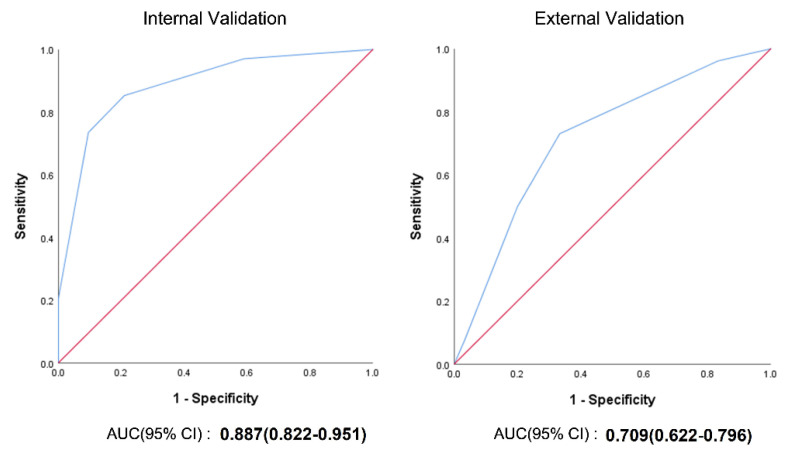
Receiver operating characteristic (ROC) curve and area under the curve (AUC) of the CoBiF score on internal and external validation.

Internal validation was conducted using the bias-corrected AUC (0.818), which demonstrated good predictability. The overall patient characteristics in the internal and external datasets are presented in Table 4. The validation cohort had a worse medical condition in terms of age and comorbidities; the original geographic areas of the two datasets were notably different. The rates of adverse outcomes, such as ICU admission and mortality, were greater in the external dataset than in the internal dataset. The AUC of the CoBiF scoring system during external validation was 0.709 (95% CI: 0.622–0.796; Figure 2).

## 5. Discussion

This study described the clinical manifestations, management, and prognosis of spontaneously developed PNx/PMEx among COVID-19 patients based on the current literature. This study included observations from various countries and reported real-world data by including institutions with limited resources. We have developed a novel CoBiF scoring system to predict in-hospital mortality among patients with COVID-19 who developed PNx/PMEx without prior IMV. Notably, we incorporated COVID-19 patient data from eight countries and evaluated their disease severity. 

Each factor included in the CoBiF scoring system seemed to incorporate the results of previous studies on the prognosis of COVID-19 patients, representing various risk factors for mortality. These factors include age, symptoms (fever, hemoptysis, dyspnea, and loss of consciousness), comorbidities (number of diseases, cancer history, and hypertension), and laboratory findings (D-dimer level, neutrophil-to-lymphocyte ratio, lactate dehydrogenase level, and bilirubin level) [14,15,16]. Fever was considered a poor prognostic sign based on an Iranian national data [17], and it was observed more commonly in COVID-19-associated PNx/PMEx than in non-COVID-19-associated PNx [5]. However, the clinical interpretation of fever needs caution since the causes of it could differ according to the patients’ population; patients with fever had more underlying medical conditions in this study. As multicollinearity was not observed between comorbidity and fever in the analysis for in-hospital mortality, we could not definitively describe possible other causes for fever. In this study, the presence of fever seemed to be more related to the severity of COVID-19 infection. Even so, clinicians should consider diverse reasons for fever depending on patients’ characteristics.

The general predictive factors for COVID-19 also seem to be effective in predicting the prognosis of COVID-19-associated PNx/PMEx because they bear similarities to the risk factors used in the CoBiF score. Since most causes of death in our study were merely suggestive of the detrimental consequences of COVID-19, the specific nature of the relationship between mortality and PNx/PMEx could not be ascertained conclusively. Therefore, the treatment approach for COVID-19-associated PNx/PMEx should be in accordance with general COVID-19 management protocols based on current knowledge.

However, clinicians should focus minutely on bilateral spontaneous PNx, which is rarely reported in other respiratory diseases. Simultaneous bilateral PNx occurs in 1.0–1.6% of all patients with PNx [18,19,20]. These patients have adverse outcomes during hospitalization and long-term mortality [21], necessitating timely intervention [20]. Specifically, PNx is considered a poor prognostic factor, which is observed mainly in patients with underlying lung disease [19]. It is unknown whether bilateral PNx occurs more commonly in COVID-19. However, bilateral pneumonia has been observed in 75–86% of hospitalized patients with COVID-19 [22,23], with the most common CT features being peripherally distributed ground-glass opacities and bilateral lung consolidation. Moreover, the extent and intensity of opacities are suggestive of a poor prognosis [24,25]. Moreover, barotrauma in patients with IMV, i.e., PNx/PMEx, occurs more frequently in COVID-19 than in other acute respiratory distress syndromes [7]. If the vulnerability of COVID-19-infected lungs causes the development of PNx/PMEx, bilateral PNx may indicate the fatal nature of COVID-19. The causal relationship and underlying pathophysiology require further investigation.

The mechanism underlying the pathogenesis of PNx/PMEx in COVID-19 remains obscure, and several hypotheses, including air leakage through the alveolar walls [26], increased vulnerability to PNx/PMEx arising from cyst formation due to severe damage induced by inflammation [27,28], and the Macklin effect as a cause for PMEx, have been postulated [29]. However, recent studies found no difference between the histopathologic findings of COVID-19 and other causes of lung injury [28,30]. Further comparative studies are needed to elucidate the specific mechanism underlying the formation of PNx/PMEx in COVID-19.

Our study has several limitations. First, although we presented data from multiple countries, the heterogeneity in their respective clinical environments and the capacity to deal with the pandemic could have impacted the outcomes. Second, since cases were collected based on the authors’ recall and retrospective review of medical records, publication bias could arise from the inclusion of a few patients with poor outcomes. Additionally, this study could not present the laboratory findings of the patients’ medical conditions as they were selectively reported by most studies; thus, disease severity in the patients included in this review could not be compared quantitatively. Despite our great efforts to contact the authors of these case reports, we were unable to contact all authors and validate patient data. Moreover, the medical management of COVID-19 in the included cases was highly heterogeneous; some patients were treated with medications that are no longer used for COVID-19 treatment. Therefore, the study findings should be interpreted cautiously in the evolving contemporary context of the COVID-19 pandemic, viz., the different dominant virus strains, vaccine availability, and innate immunity level. 

## 6. Conclusions

This study is important because it comprehensively reviewed cases of spontaneous PNx/PMEx in COVID-19 and presented a numerically measured prediction model. This novel CoBiF scoring system can be applied in other clinical settings, assist clinicians in identifying patients at high risk of mortality, and facilitate more prompt management. A further improved scoring system can be devised after the accrual of more evidence and research on spontaneous PNx/PMEx in COVID-19.

## Figures and Tables

**Table 1:** Clinical characteristics and outcomes of patients according to the type of disease.

Factor	Total	PMEx	PNx ± PMEx	*p*-Value
	*n* = 151	*n* = 43	*n* = 108	
Age, years	56.0 [40.5, 67.5]	54.0 [38.0, 64.5]	57.5 [41.0, 68.0]	0.340
Sex				0.462
Female	23 (15.2)	8/43 (18.6)	15/108 (13.9)	
Male	128 (84.8)	35/43 (81.4)	93/108 (86.1)	
Comorbidity				
Comorbidity present	80/147 (54.4)	26/41 (63.4)	54/106 (50.9)	0.199
Obesity	17/143 (11.9)	9/40 (22.5)	8/103 (7.8)	0.021
Diabetes mellitus	17/143 (11.9)	5/40 (12.5)	12/103 (11.7)	1
Hypertension	39/143 (27.3)	10/40 (25.0)	29/103 (28.2)	0.835
Respiratory disease	16/143 (11.2)	3/40 (7.5)	13/103 (12.6)	0.557
COVID-19-targeted treatments				
Steroid	68/116 (58.6)	25/35 (71.4)	43/81 (53.1)	0.100
Convalescent plasma	7/87 (8.0)	0/29 (0.0)	7/58 (12.1)	0.090
Intravenous immunoglobulin	4/90 (4.4)	2/30 (6.7)	2/60 (3.3)	0.598
Lopinavir/ritonavir	10/90 (11.1)	4/30 (13.3)	6/60 (10.0)	0.726
Remdesivir	19/92 (20.7)	7/30 (23.3)	12/62 (19.4)	0.784
Tocilizumab	13/90 (14.4)	3/30 (10.0)	10/60 (16.7)	0.532
Clinical manifestations				
Chest pain	55/123 (44.7)	11/34 (32.4)	44/89 (49.4)	0.107
Cough	45/134 (33.6)	13/40 (32.5)	32/94 (34.0)	1
Fever	50/142 (35.2)	1740 (42.5)	33/102 (32.4)	0.329
Dyspnea	114/136 (83.8)	33/40 (82.5)	81/96 (84.4)	0.801
Oxygen saturation (room air), %	85.0 [80.0, 91.0]	85.5 [81.8, 88.8]	84.5 [80.0, 91.0]	0.752
Subcutaneous emphysema	40/143 (28.0)	13/42 (31.0)	27/101 (26.7)	0.683
Chest CT findings				
Emphysema	11/137 (8.0)	5/36 (13.9)	6/101 (5.9)	0.157
Ground-glass opacity	106/130 (81.5)	27/31 (87.1)	79/99 (79.8)	0.436
Pleural effusion	11/136 (8.1)	2/35 (5.7)	9/101 (8.9)	0.728
Treatments				
Conservative care	73/135 (54.1)	33/35 (94.3)	40/100 (40.0)	<0.001
Chest tube insertion	82/131 (62.6)	0/31 (0.0)	82/100 (82.0)	<0.001
Surgery	8/139 (5.8)	0/37 (0.0)	8/102 (7.8)	0.109
Non-invasive ventilation	9/134 (6.7)	5/34 (14.7)	4/100 (4.0)	0.046
Invasive mechanical ventilation	30/145 (20.7)	9/40 (22.5)	21/105 (20.0)	0.819
Outcome				
Hospital stay, days	13.0 [6.0, 19.0]	14.0 [9.5, 15.8]	13.0 [6.0, 19.0]	0.975
ICU admission	51/138 (37.0)	14/36 (38.9)	37/102 (36.3)	0.842
Mortality	35/151 (23.2)	9/43 (20.9)	26/108 (24.1)	0.831

All data are presented as *n* (%), *n*/N (%), or median [interquartile range]. COVID-19, coronavirus disease 2019; CT, computed tomography; ICU, intensive care unit; PMEx, pneumomediastinum; PNx, pneumothorax. Close monitoring and/or supplemental oxygen support through a nasal cannula and reservoir mask.

**Table 2:** Clinical characteristics and outcomes of patients with PNx/PMEx according to outcome.

Factor	Survivors	Non-Survivors	*p*-Value
	*n* = 116	*n* = 35	
Age, years	52.5 [38.0, 65.0]	67.0 [55.0, 75.0]	<0.001
Sex			0.597
Female	19/116 (16.4)	4/35 (11.4)	
Male	97/116 (83.6)	31/35 (88.6)	
Comorbidity			
Comorbidity present	52/112 (46.4)	28/35 (80.0)	<0.001
Obesity	8/108 (7.4)	9/35 (25.7)	0.007
Diabetes mellitus	11/108 (10.2)	6/35 (17.1)	0.366
Hypertension	23/108 (21.3)	16/35 (45.7)	0.008
Respiratory disease	13/108 (12.0)	3/35 (8.6)	0.761
COVID-19 targeted treatments			
Steroid	45/88 (51.1)	23/28 (82.1)	0.004
Convalescent plasma	6/63 (9.5)	1/24 (4.2)	0.668
Intravenous immunoglobulin	4/66 (6.1)	0/24 (0.0)	0.570
Lopinavir/ritonavir	8/66 (12.1)	2/24 (8.3)	1
Remdesivir	10/68 (14.7)	9/24 (37.5)	0.037
Tocilizumab	11/66 (16.7)	2/24 (8.3)	0.501
Hydroxychloroquine ^*^	20/67 (29.9)	7/24 (29.2)	1
Clinical manifestations			
Chest pain	49/95 (51.6)	6/28 (21.4)	0.005
Cough	29/102 (28.4)	16/32 (50.0)	0.032
Fatigue	3/95 (3.2)	3/28 (10.7)	0.131
Fever	21/108 (19.4)	29/34 (85.3)	<0.001
Dyspnea	83/104 (79.8)	31/32 (96.9)	0.026
Oxygen saturation (room air), %	85.0 [80.0, 91.0]	84.0 [75.0, 89.0]	0.275
PNx/PMEx characteristics			
Type of disease			0.038
PMEx	34/116 (29.3)	9/35 (25.7)	
PNx	63/116 (54.3)	13/35 (37.1)	
Both	19/116 (16.4)	13/35 (37.1)	
PNx location			0.029
Bilateral	9/80 (11.2)	8/22 (36.4)	
Left	32/80 (40.0)	7/22 (31.8)	
Right	39/80 (48.8)	7/22 (31.8)	
Tension PNx	15/104 (14.4)	3/29 (10.3)	0.762
Subcutaneous emphysema	28/110 (25.5)	12/33 (36.4)	0.269
Chest CT findings			
Emphysema	7/107 (6.5)	4/30 (13.3)	0.256
Ground-glass opacity	82/102 (80.4)	24/28 (85.7)	0.596
Pleural effusion	6/106 (5.7)	5/30 (16.7)	0.065
Visible bullae	15/106 (14.2)	1/30 (3.3)	0.195
Treatments			
Conservative care	56/105 (53.3)	17/30 (56.7)	0.837
Chest tube insertion	65/104 (62.5)	17/27 (63.0)	1
Surgery	8/108 (7.4)	0/31 (0.0)	0.199
Non-invasive ventilation	6/104 (5.8)	3/30 (10.0)	0.418
Invasive mechanical ventilation	11/111 (9.9)	19/34 (55.9)	<0.001
Outcome			
Chest tube indwelling time, days	5.5 [3.0, 8.8]	10.0 [6.0, 10.5]	0.714
Hospital stay, days	12.0 [6.0, 25.0]	14.0 [9.3, 18.0]	0.938
ICU admission	23/107 (21.5)	28/31 (90.3)	<0.001

All data are presented as *n* (%), *n*/N (%), or median [interquartile range]. COVID-19, coronavirus disease 2019; CT, computed tomography; ICU, intensive care unit; PMEx, pneumomediastinum; PNx, pneumothorax. * Hydroxychloroquine was used during the early period of the pandemic when sufficient evidence was lacking. It should not be used anymore. Close monitoring and/or supplemental oxygen support through a nasal cannula and reservoir mask.

**Table 3:** Risk factor analysis for in-hospital mortality among patients with COVID-19-associated PNx/PMEx.

Univariate			Multivariable	
Factor	OR (95% CI)	*p*-Value	OR (95% CI)	*p*-Value
Male (ref. Female)	1.52 (0.48–4.80)	0.477		
Comorbidity present	4.35 (1.72–11.1)	0.002	3.87 (1.27–11.9)	0.018
Diabetes mellitus	1.82 (0.62–5.36)	0.274		
Hypertension	2.80 (1.19–6.57)	0.018	1.22 (0.32–4.61)	0.772
Bilateral PNx	3.67 (1.35–9.95)	0.009	4.86 (1.08–21.9)	0.039
Tension PNx	0.69 (0.18–2.55)	0.572		
Subcutaneous emphysema	1.67 (0.73–3.83)	0.222		
Type of diseases (ref. PMEx)				
PNx	0.78 (0.30–2.01)	0.612		
PNx with PMEx	2.58 (0.93–7.16)	0.068		
Symptoms–fever	24.0 (8.31–69.5)	<0.001	24.1 (7.75–74.6)	<0.001
Symptoms–cough	2.52 (1.11–5.69)	0.026	1.13 (0.35–3.65)	0.838
Symptoms–dyspnea	7.84 (1.01–60.8)	0.049	3.25 (0.33–31.9)	0.312
CT findings–GGO	1.46 (0.46–4.70)	0.522		
CT findings–emphysema	2.20 (0.60–8.08)	0.236		
CT findings–pleural effusion	3.33 (0.94–11.80)	0.062		

CI, confidence interval; COVID-19, coronavirus disease 2019; CT, computed tomography; GGO, ground-glass opacity; OR, odds ratio; PMEx, pneumomediastinum; PNx, pneumothorax; ref: reference; Tx, treatments.

**Table 4:** Clinical characteristics and outcomes of patients: comparison of the training and validation cohorts.

	Training Cohort ^¶^	Validation Cohort ^⁂^	*p*-Value
Factor	*n* = 151	*n* = 133	
Age, years	56.0 [40.5, 67.5]	64.0 [51.0, 72.0]	0.004
Sex			<0.001
Female	23/151 (15.2)	49/133 (36.8)	
Male	128/151 (84.8)	84/133 (63.2)	
Comorbidities present	80/147 (54.4)	101/133 (75.9)	<0.001
Diabetes mellitus	17/143 (11.9)	36/133 (27.1)	0.002
Obesity	17/143 (11.9)	32/133 (24.1)	0.011
Hypertension	39/143 (27.3)	69/133 (51.9)	<0.001
Respiratory diseases	16/143 (11.2)	19/133 (14.3)	0.473
Geographical area of data sources			<0.001
Africa	3/151 (2.0)	0/133 (0.0)	
Asia	41/151 (27.2)	100/133 (75.2)	
Europe	59/151 (39.1)	33/133 (24.8)	
North America	42/151 (27.8)	0/133 (0.0)	
South America	6/151 (4.0)	0/133 (0.0)	
Clinical scenario			<0.001
Initial presentation	68/151 (45.0)	56/133 (42.1)	
During hospitalization	65/151 (43.9)	77/133 (57.9)	
Recent recovery from COVID-19	18/151 (12.2)	0/133 (0.0)	
Presenting symptom of PNx/PMEx			
Chest pain	55/123 (44.7)	48/128 (37.5)	0.251
Cough	45/134 (33.6)	86/128 (67.2)	<0.001
Dyspnea	114/136 (83.8)	106/128 (82.8)	0.870
Fever	50/142 (35.2)	76/133 (57.1)	<0.001
Oxygen saturation at room air	85.0 [80.0, 91.0]	87.0 [80.0, 90.0]	0.784
Type of PNx/PMEx			0.004
PMEx alone	43/151 (28.5)	19/133 (14.3)	
PNx alone	76/151 (50.3)	69/133 (51.9)	
PNx + PMEx	32/151 (21.2)	45/133 (33.8)	
PNX/PMEx-related characteristics			
Subcutaneous emphysema	40/143 (28.0)	62/133 (46.6)	0.002
Tension PNx	18/133 (13.5)	9/103 (8.7)	0.305
PNx location			0.282
Bilateral	17/102 (16.7)	26/107 (24.3)	
Left	39/102 (38.2)	43/107 (40.2)	
Right	46/102 (45.1)	38/107 (35.5)	
Radiological findings on chest CT			
Visible Bullae	16/136 (11.8)	8/128 (6.2)	0.137
Ground-glass opacity	106/130 (81.5)	102/128 (79.7)	0.754
Pleural effusion	11/136 (8.1)	14/128 (10.9)	0.529
Treatment			
Conservative care	73/135 (54.1)	45/129 (34.9)	0.002
Chest tube drainage	82/131 (62.6)	97/133 (72.9)	0.087
Surgery	8/139 (5.8)	2/128 (1.6)	0.106
Outcome			
Hospital stay, days	13.0 [6.0, 19.0]	17.0 [9.0, 26.0]	0.016
ICU admission	51/138 (37.0)	98/133 (73.7)	<0.001
Mortality	35/151 (23.2)	83/133 (62.4)	<0.001

All data are presented as *n* (%), *n*/N (%), or median [interquartile range]. COVID-19, coronavirus disease 2019; CT, computed tomography; ICU, intensive care unit; PMEx, pneumomediastinum; PNx, pneumothorax. **^¶^** Data from case series and case reports based on the systematic review. **^⁂^** Patients from the International COVID-19 Pneumothorax Working Group (ICP-WG).

## Data Availability

The data that support the findings of this study are available from the corresponding author upon reasonable request.

## Supplementary Materials

The following supporting information can be downloaded at: https://www.mdpi.com/article/10.3390/jcm11237132/s1. Supplementary Table S1. Checklist summarizing compliance with PRISMA guidelines. Supplementary Table S2. Detailed search strategy according to database. Supplementary Table S3. The Lists of included studies [6,31,32,33,34,35,36,37,38,39,40,41,42,43,44,45,46,47,48,49,50,51,52,53,54,55,56,57,58,59,60,61,62,63,64,65,66,67,68,69,70,71,72,73,74,75,76,77,78,79,80,81,82,83,84,85,86,87,88,89,90,91,92,93,94,95,96,97,98,99,100,101,102,103,104,105,106,107,108,109,110,111,112,113,114,115,116,117,118,119,120,121,122,123,124,125,126,127,128,129,130,131,132]. Supplementary Table S4. Clinical characteristic and outcome of patients according to presenting clinical scenarios. Supplementary Table S5. The Causes of death among deceased patients (*n* = 35). Supplementary Figure S1. PRISMA flow diagram of selection processes.

## Abbreviations

Coronavirus disease 2019	COVID-19
Pneumothorax	PNx
Pneumomediastinum	PMEx
invasive mechanical ventilation	IMV
International COVID-19 Pneumothorax Working Group	ICP-WG
Preferred Reporting Items for Systematic Reviews and Meta-Analyses Protocols	PRISMA-P
computed tomography	CT
intensive care unit	ICU
odds ratio	OR
area under the curve	AUC
Statistical Package for the Social Sciences	SPSS
confidence interval	CI
