# Supplementary material for: Prognosis of Spontaneous Pneumothorax/Pneumomediastinum in Coronavirus Disease 2019: The CoBiF Score
Source: J Clin Med. 2022 Nov 30;11(23):7132. doi: 10.3390/jcm11237132 (PMC9739102; doi:10.3390/jcm11237132)

Supplementary Table S1. Checklist summarizing compliance with PRISMA guidelines.

Section/topic	#	Checklist item	Reported on page #
**TITLE**			
Title	1	Identify the report as a systematic review, meta-analysis, or both.	1
**ABSTRACT**			
Structured summary	2	Provide a structured summary including, as applicable: background; objectives; data sources; study eligibility criteria, participants, and interventions; study appraisal and synthesis methods; results; limitations; conclusions and implications of key findings; systematic review registration number.	5-6
**INTRODUCTION**			
Rationale	3	Describe the rationale for the review in the context of what is already known.	7
Objectives	4	Provide an explicit statement of questions being addressed with reference to participants, interventions, comparisons, outcomes, and study design (PICOS).	7-8
**METHODS**			
Protocol and registration	5	Indicate if a review protocol exists, if and where it can be accessed (e.g., Web address), and, if available, provide registration information including registration number.	8
Eligibility criteria	6	Specify study characteristics (e.g., PICOS, length of follow-up) and report characteristics (e.g., years considered, language, publication status) used as criteria for eligibility, giving rationale.	8
Information sources	7	Describe all information sources (e.g., databases with dates of coverage, contact with study authors to identify additional studies) in the search and date last searched.	8
Search	8	Present full electronic search strategy for at least one database, including any limits used, such that it could be repeated.	8 (supplementary table E2)
Study selection	9	State the process for selecting studies (i.e., screening, eligibility, included in systematic review, and, if applicable, included in the meta-analysis).	8-9
Data collection process	10	Describe method of data extraction from reports (e.g., piloted forms, independently, in duplicate) and any processes for obtaining and confirming data from investigators.	9
Data items	11	List and define all variables for which data were sought (e.g., PICOS, funding sources) and any assumptions and simplifications made.	9

Risk of bias in individual studies	12	Describe methods used for assessing risk of bias of individual studies (including specification of whether this was done at the study or outcome level), and how this information is to be used in any data synthesis.	N/A
Summary measures	13	State the principal summary measures (e.g., risk ratio, difference in means).	9
Synthesis of results	14	Describe the methods of handling data and combining results of studies, if done, including measures of consistency (e.g., I^2) for each meta-analysis.	9-11
**Section/topic**	**#**	**Checklist item**	**Reported on page #**
Risk of bias across studies	15	Specify any assessment of risk of bias that may affect the cumulative evidence (e.g., publication bias, selective reporting within studies).	N/A
Additional analyses	16	Describe methods of additional analyses (e.g., sensitivity or subgroup analyses, meta-regression), if done, indicating which were pre-specified.	N/A
**RESULTS**			
Study selection	17	Give numbers of studies screened, assessed for eligibility, and included in the review, with reasons for exclusions at each stage, ideally with a flow diagram.	Supplementary Figure E1
Study characteristics	18	For each study, present characteristics for which data were extracted (e.g., study size, PICOS, follow-up period) and provide the citations.	Supplementary Table E3
Risk of bias within studies	19	Present data on risk of bias of each study and, if available, any outcome level assessment (see item 12).	N/A
Results of individual studies	20	For all outcomes considered (benefits or harms), present, for each study: (a) simple summary data for each intervention group (b) effect estimates and confidence intervals, ideally with a forest plot.	Table 1-2<br>Supplementary Table E3
Synthesis of results	21	Present results of each meta-analysis done, including confidence intervals and measures of consistency.	12-17<br>Table 1-3<br>Supplementary Table E4-6
Risk of bias across studies	22	Present results of any assessment of risk of bias across studies (see Item 15).	N/A
Additional analysis	23	Give results of additional analyses, if done (e.g., sensitivity or subgroup analyses, meta-regression [see Item 16]).	N/A
**DISCUSSION**			
Summary of evidence	24	Summarize the main findings including the strength of evidence for each main outcome; consider their relevance to key groups (e.g., healthcare providers, users, and policy makers).	22-24
Limitations	25	Discuss limitations at study and outcome level (e.g., risk of bias), and at review-level (e.g., incomplete retrieval of identified research, reporting bias).	24

Conclusions	26	Provide a general interpretation of the results in the context of other evidence, and implications for future research.	25
**FUNDING**			
Funding	27	Describe sources of funding for the systematic review and other support (e.g., supply of data); role of funders for the systematic review.	26

From: Moher D, Liberati A, Tetzlaff J, Altman DG, The PRISMA Group (2009). Preferred Reporting Items for Systematic Reviews and Meta-Analyses: The PRISMA Statement. PLoS Med 6(6): e1000097. doi:10.1371/journal.pmed1000097

Supplementary Table S2. Detailed search strategy according to database

#1. Search Strategy (21 December 2021)

**PubMed Search Strategy (285 hits)**<br><br>Years/Issue Searched: 2020 to 2021<br>Search date: 21 December 2021<br>(“sars-cov-2” OR “covid 19” OR “covid-19” OR “covid19” OR “human coronavirus 2019” OR “sars coronavirus 2” OR “wuhan coronavirus”) AND (“pneumothorax” OR “pneumomediastinum” OR “pneumo*”) Filters: English, from 2020 – 2021
**Embase Search Strategy (245 hits)**<br>#1. ‘human coronavirus 2019 vaccine’ OR ‘sars coronavirus 2 vaccine’ OR ‘wuhan coronavirus vaccine’<br>#2. ‘pneumothorax’ OR ‘pneumomediastinum’<br>#3. #1 AND #2 AND [english]/lim AND [2020-2021]/py
**Scopus Search Strategy (296 hits)**<br><br>Years/Issue Searched: 2020 to 2021<br>Search date: 21 December 2021<br>(“sars-cov-2” OR “covid 19” OR “covid-19” OR “covid19” OR “human coronavirus 2019” OR “sars coronavirus 2” OR “wuhan coronavirus”) AND (“pneumothorax” OR “pneumomediastinum” OR “pneumo*”) AND (LIMT-TO (PUBYEAR,2021) OR LIMT-TO (PUBYEAR,2020)) AND (LIMIT-TO (LANGUAGE, “English”))

Supplementary Table S3. The lists of included studies

References of included studies
1 Amoah K, Gunasekaran K, Rahi MS, Buscher MG. A case of secondary tension pneumothorax in COVID-19 pneumonia in a patient with no prior history of lung disease. SAGE Open Med Case Rep 2020; 8: 2050313X20967504.<br>2 Caviezel C, Weiss L, Haessig G, et al. Case report of sequential bilateral spontaneous pneumothorax in a never-ventilated, lung-healthy COVID-19-patient. Int J Surg Case Rep 2020; 75: 441–5.<br>3 Ferreira JG, Rapparini C, Gomes BM, Pinto LAC, Freire MS da SE. Pneumothorax as a late complication of COVID-19. Rev Inst Med Trop Sao Paulo 2020; 62: e61.<br>4 Gillespie M, Dincher N, Fazio P, Okorji O, Finkle J, Can A. Coronavirus disease 2019 (COVID-19) complicated by Spontaneous Pneumomediastinum and Pneumothorax. Respir Med Case Rep 2020; 31: 101232.<br>5 Kolani S, Houari N, Haloua M, et al. Spontaneous pneumomediastinum occurring in the SARS-COV-2 infection. IDCases 2020; 21: e00806.<br>6 Mimouni H, Diyas S, Ouachaou J, et al. Spontaneous Pneumomediastinum Associated with COVID-19 Pneumonia. Case Rep Med 2020; 2020: 4969486.<br>7 Muhammad AI, Boynton EJ, Naureen S. COVID-19 with bilateral pneumothoraces- case report. Respir Med Case Rep 2020; 31: 101254.<br>8 Rehman T, Josephson G, Sunbuli M, Chadaga AR. Spontaneous Pneumothorax in an Elderly Patient With Coronavirus Disease (COVID-19) Pneumonia. Ochsner J 2020; 20: 343–5.<br>9 Shan S, Guangming L, Wei L, Xuedong Y. Spontaneous pneumomediastinum, pneumothorax and subcutaneous emphysema in COVID-19: case report and literature review. Rev Inst Med Trop Sao Paulo 2020; 62: e76.<br>10 Ahluwalia AS, Qarni T, Narula N, Sadiq W, Chalhoub MN. Bilateral pneumothorax as possible atypical presentation of coronavirus disease 2019 (COVID-19). Respiratory Medicine Case Reports 2020; 31: 101217.<br>11 Wang W, Gao R, Zheng Y, Jiang L. COVID-19 with spontaneous pneumothorax,pneumomediastinum and subcutaneous emphysema. J Travel Med 2020; : taaa062.<br>12 Liu K, Zeng Y, Xie P, et al. COVID-19 with cystic features on computed tomography: A case report. Medicine (Baltimore) 2020; 99: e20175.<br>13 Rohailla S, Ahmed N, Gough K. SARS-CoV-2 infection associated with spontaneous pneumothorax. CMAJ 2020; 192: E510.<br>14 Bellini D, Lichtner M, Vicini S, Rengo M, Ambrogi C, Carbone I. Spontaneous pneumomediastinum as the only CT finding in an asymptomatic adolescent positive for COVID-19. BJR Case Rep 2020; 6: 20200051.<br>15 Flower L, Carter J-PL, Rosales Lopez J, Henry AM. Tension pneumothorax in a patient with COVID-19. BMJ Case Rep 2020; 13: e235861.<br>16 Ucpinar BA, Sahin C, Yanc U. Spontaneous pneumothorax and subcutaneous emphysema in COVID-19 patient: Case report. J Infect Public Health 2020; 13: 887–9.<br>17 Goldman N, Ketheeswaran B, Wilson H. COVID-19-associated pneumomediastinum. Clin Med (Lond) 2020; 20: e91–2.<br>18 Wegner U, Jeffery G, Abrajan O, Sampablo I, Singh C. Spontaneous Pneumomediastinum Associated With SARS-CoV-2: Infrequent Complication of the Novel Disease. Cureus 2020; 12: e9189.<br>19 Giné C, Laín A, García L, López M. Thoracoscopic Bullectomy for Persistent Air Leak in a 14-Year-Old Child with COVID-19 Bilateral Pulmonary Disease. J Laparoendosc Adv Surg Tech A 2020; 30: 935–8.<br>20 Khurram R, Johnson FTF, Naran R, Hare S. Spontaneous tension pneumothorax and acute pulmonary emboli in a patient with COVID-19 infection. BMJ Case Rep 2020; 13: e237475.

21 Kong N, Gao C, Xu M-S, Xie Y-L, Zhou C-Y. Spontaneous pneumomediastinum in an elderly COVID-19 patient: A case report. World J Clin Cases 2020; 8: 3573–7.
22 Sonia F, Kumar M. A Complication of Pneumothorax and Pneumomediastinum in a Non-Intubated Patient With COVID-19: A Case Report. Cureus 2020; 12: e10044.
23 Alhakeem A, Khan MM, Al Soub H, Yousaf Z. Case Report: COVID-19-Associated Bilateral Spontaneous Pneumothorax-A Literature Review. Am J Trop Med Hyg 2020; 103: 1162–5.
24 Quincho-Lopez A, Quincho-Lopez DL, Hurtado-Medina FD. Case Report: Pneumothorax and Pneumomediastinum as Uncommon Complications of COVID-19 Pneumonia-Literature Review. Am J Trop Med Hyg 2020; 103: 1170–6.
25 Yasukawa K, Vamadevan A, Rollins R. Bulla Formation and Tension Pneumothorax in a Patient with COVID-19. Am J Trop Med Hyg 2020; 103: 943–4.
26 Salah O, Faisal M, Alshahwani I, Elhiday A. Bilateral Hemopneumothorax in COVID-19. Cureus 2020; 12: e10314.
27 Chen X, Zhang G, Tang Y, Peng Z, Pan H. The coronavirus diseases 2019 (COVID-19) pneumonia with spontaneous pneumothorax: a case report. BMC Infect Dis 2020; 20: 662.
28 Fahad AM, Mohammad AA, Al-Khalidi HA, Alshewered AS. Spontaneous pneumothorax as a complication in COVID-19 male patient: A case report. Clin Case Rep 2020; 8: 3116–9.
29 Fan Q, Pan F, Yang L. Spontaneous pneumothorax and subpleural bullae in a patient with COVID-19: a 92-day observation. Eur J Cardiothorac Surg 2020; 58: 858–60.
30 Bellini R, Salandini MC, Cuttin S, Mauro S, Scarpazza P, Cotsoglou C. Spontaneous pneumothorax as unusual presenting symptom of COVID-19 pneumonia: surgical management and pathological findings. J Cardiothorac Surg 2020; 15: 310.
31 Hameed M, Jamal W, Yousaf M, et al. Pneumothorax In Covid-19 Pneumonia: A case series. Respir Med Case Rep 2020; 31: 101265.
32 Berhane S, Tabor A, Sahu A, Singh A. Development of bullous lung disease in a patient with severe COVID-19 pneumonitis. BMJ Case Rep 2020; 13: e237455.
33 Manna S, Maron SZ, Cedillo MA, et al. Spontaneous subcutaneous emphysema and pneumomediastinum in non-intubated patients with COVID-19. Clin Imaging 2020; 67: 207–13.
34 Perice L, Roit Z, Llovera I, Flanagan-Kundle MG. Spontaneous Pneumothorax as a Complication of COVID-19 Pneumonia: A Case Report. Clin Pract Cases Emerg Med 2020; 4: 521–3.
35 Zayet S, Klopfenstein T, Mezher C, Gendrin V, Conrozier T, Ben Abdallah Y. Coronavirus disease 2019 with spontaneous pneumothorax, pneumomediastinum and subcutaneous emphysema, France. New Microbes New Infect 2020; 38: 100785.
36 Singh A, Bass J, Lindner DH. Rare Complication of Pneumomediastinum and Pneumopericardium in a Patient with COVID-19 Pneumonia. Case Rep Pulmonol 2020; 2020: 8845256.
37 Yamaya T, Baba T, Hagiwara E, et al. Pneumothorax in a COVID-19 Pneumonia Patient without Underlying Risk Factors. Intern Med 2020; 59: 2921–5.
38 Tucker L, Patel S, Vatsis C, et al. Pneumothorax and Pneumomediastinum Secondary to COVID-19 Disease Unrelated to Mechanical Ventilation. Case Rep Crit Care 2020; 2020: 6655428.
39 Ayazi S, Zebarjadi J, Grubic AD, Tahmasbi H, Ayazi K, Jobe BA. Pneumothorax as the presenting manifestation of COVID-19. J Thorac Dis 2020; 12: 7488–93.
40 Brogna B, Bignardi E, Salvatore P, et al. Unusual presentations of COVID-19 pneumonia on CT scans with spontaneous pneumomediastinum and loculated pneumothorax: A report of two cases and a review of the literature. Heart Lung 2020; 49: 864–8.
41 López Vega JM, Parra Gordo ML, Diez Tascón A, Ossaba Vélez S. Pneumomediastinum and spontaneous pneumothorax as an extrapulmonary complication of COVID-19 disease. Emerg Radiol 2020; 27: 727–30.
42 Spiro JE, Sisovic S, Ockert B, Böcker W, Siebenbürger G. Secondary tension pneumothorax in a COVID-19 pneumonia patient: a case report. Infection 2020; 48: 941–4.
43 Urigo C, Soin S, Sahu A. Spontaneous pneumomediastinum as a complication of a COVID-19 related pneumonia: case report and review of literature. Radiol Case Rep 2020; 15: 2577–81.

44 Sanivarapu RR, Farraj K, Sayedy N, Anjum F. Rapidly developing large pneumatocele and spontaneous pneumothorax in SARS-CoV-2 infection. Respir Med Case Rep 2020; 31: 101303.
45 Vahidirad A, Jangjoo A, Ghelichli M, Arian Nia A, Zandbaf T. Tension pneumothorax in patient with COVID-19 infection. Radiol Case Rep 2020; 16: 358–60.
46 Elhakim TS, Abdul HS, Pelaez Romero C, Rodriguez-Fuentes Y. Spontaneous pneumomediastinum, pneumothorax and subcutaneous emphysema in COVID-19 pneumonia: a rare case and literature review. BMJ Case Rep 2020; 13: e239489.
47 Afrazi A, Garcia-Rodriguez S, Maloney JD, Morgan CT. Cavitary lung lesions and pneumothorax in a healthy patient with active coronavirus-19 (COVID-19) viral pneumonia. Interact Cardiovasc Thorac Surg 2020; 32: 150–2.
48 Capleton P, Ricketts W, Lau K, et al. Pneumothorax and Pneumatocoele Formation in a Patient with COVID-19: a Case Report. SN Compr Clin Med 2021; 3: 269–72.
49 Tamaskani N, Khandashpour M, Livani S. Can spontaneous pneumothorax be resolved in COVID-19 without hospital care? A case report. Caspian J Intern Med 2021; 12: S368–70.
50 González-Pacheco H, Gopar-Nieto R, Jiménez-Rodríguez G-M, Manzur-Sandoval D, Sandoval J, Arias-Mendoza A. Bilateral spontaneous pneumothorax in SARS-CoV-2 infection: A very rare, life-threatening complication. Am J Emerg Med 2021; 39: 258.e1-258.e3.
51 Nobre Pereira M, Blanco R, Areias V. Pneumomediastinum: An Uncommon Complication of COVID-19 Pneumonia. Arch Bronconeumol 2021; 57: 68.
52 Szewczyk J, Adkinson BC, Akkineni S, Nguyen DM, Arias SA, Villamizar NR. Endobronchial valves: a bridge to definitive surgical management in COVID-19 recurrent pneumothorax. J Thorac Dis 2021; 13: 411–3.
53 Jatoi TA, Khan AA, Mohiuddin O, Choudhry MS, Yasmin F, Jalees S. Spontaneous pneumomediastinum and subcutaneous emphysema in a non-intubated COVID-19 patient: a case report. Pan Afr Med J 2021; 38: 37.
54 Nunna K, Braun AB. Development of a large spontaneous pneumothorax after recovery from mild COVID-19 infection. BMJ Case Rep 2021; 14: e238863.
55 Marza AM, Petrica A, Buleu FN, Mederle OA. Case Report: Massive Spontaneous Pneumothorax—A Rare Form of Presentation for Severe COVID-19 Pneumonia. Medicina (Kaunas) 2021; 57: 82.
56 Janssen J, Kamps MJA, Joosten TMB, Barten DG. Spontaneous pneumomediastinum in a male adult with COVID-19 pneumonia. The American Journal of Emergency Medicine 2021; 40: 228.e3-228.e5.
57 Nalewajska M, Feret W, Wojczyński Ł, Witkiewicz W, Wiśniewska M, Kotfis K. Spontaneous Pneumothorax in COVID-19 Patients Treated with High-Flow Nasal Cannula outside the ICU: A Case Series. Int J Environ Res Public Health 2021; 18: 2191.
58 Doğan Bİ, Mahleç Anar C, Sertoğullarından B, Turan MO. Pneumomediastinum as a complication of COVID-19 disease: A case report. Tuberk Toraks 2021; 69: 94–7.
59 Rafiee MJ, Babaki Fard F, Samimi K, Rasti H, Pressacco J. Spontaneous pneumothorax and pneumomediastinum as a rare complication of COVID-19 pneumonia: Report of 6 cases. Radiol Case Rep 2021; 16: 687–92.
60 Rashedi S, Mardani M, Fooladgar M, Aliannejad R. Spontaneous pneumomediastinum, pneumopericardium, pneumothorax, and subcutaneous emphysema in a patient with COVID-19. Radiol Case Rep 2021; 16: 1158–61.
61 Hamad A-MM, El-Saka HA. Post COVID-19 large pneumatocele: clinical and pathological perspectives. Interact Cardiovasc Thorac Surg 2021; : ivab072.
62 Cancelliere A, Procopio G, Mazzitelli M, et al. A case report of pneumomediastinum in a COVID-19 patient treated with high-flow nasal cannula and review of the literature: Is this a "spontaneous" complication? Clin Case Rep 2021; 9: e04007.
63 Bosher O, Syed MA, Bikmalla S. Favourable outcome after a delayed complication secondary to COVID-19. BMJ Case Rep 2021; 14: e241049.
64 Hua DT, Shah F, Perez-Corral C. A case of spontaneous pneumomediastinum in a patient with severe SARS-CoV-2 and a review of the literature. SAGE Open Med Case Rep 2021; 9:

2050313X211010021.
65 Belarbi Z, Brem FL, Nasri S, Imane S, Noha EO. An uncommon presentation of COVID-19: concomitant acute pulmonary embolism, spontaneous tension pneumothorax, pneumomediastinum and subcutaneous emphysema (a case report). Pan Afr Med J 2021; 39: 26.
66 Heijboer F, Oswald L, Cretier S, Braunstahl G-J. Pneumomediastinum in a patient with COVID-19 due to diffuse alveolar damage. BMJ Case Rep 2021; 14: e242527.
67 Pimenta I, Varudo R, Lança S, Gonzalez FA. Exuberant spontaneous pneumothorax, pneumomediastinum, pneumopericardium and subcutaneous emphysema in COVID-19 pneumonia. BMJ Case Rep 2021; 14: e243861.
68 Marzocchi G, Vassallo A, Monteduro F. Spontaneous pneumothorax as a delayed complication after recovery from COVID-19. BMJ Case Rep 2021; 14: e243578.
69 Buonsenso D, Gatto A, Graglia B, et al. Early spontaneous pneumothorax, pneumomediastinum and pneumorrhachis in an adolescent with SARS-CoV-2 infection. Eur Rev Med Pharmacol Sci 2021; 25: 4413–7.
70 Cherian A, Jha AK, Padala SRAN, Senthilnathan M. Unusual complications of spontaneous pneumomediastinum and subcutaneous emphysema in patients with SARS-CoV-2 infection: A case report. Indian J Anaesth 2021; 65: 483–6.
71 Montgomery AB, Finck C. Spontaneous hemopneumothorax in an adolescent with COVID-19. Journal of Pediatric Surgery Case Reports 2021; 69: 101852.
72 Essa RA, Ahmed SK, Bapir DH, Abubakr CP. Subcutaneous emphysema and spontaneous pneumomediastinum in non-intubated COVID-19 patient: Presenting unusual case report. Int J Surg Case Rep 2021; 84: 106071.
73 Jafari R, Cegolon L, Masghsoudi H, et al. Simultaneous Giant cavity pulmonary lesion and pneumothorax following COVID-19 pneumonia. Radiol Case Rep 2021; 16: 2534–6.
74 Khan HH, Witkowski A, Clark JA, Mata A. A 17-Year-Old Girl with a Recent History of Marijuana Use Presented with Pneumomediastinum and Pneumopericardium and Tested Positive for SARS-CoV-2 Infection on Hospital Admission. Am J Case Rep 2021; 22: e931800-1-e931800-5.
75 Mohamed A. Tension pneumothorax complicating COVID-19 pneumonia. Clin Case Rep 2021; 9: e04342.
76 Jafari R, Cegolon L, Dehghanpoor F, Javanbakht M, Tabatabaei SMH. Typical Covid-19 case with primary pneumomediastinum in a 37 year old male. Radiol Case Rep 2021; 16: 2286–8.
77 Ramezani R, Jafari F, Fahami Y, Pakniyat A, Rad MG. A case report of pneumomediastinum and subcutaneous emphysema associated with pandemic COVID-19 in a 43-year-old man. Clin Imaging 2021; 76: 74–6.
78 Protrka MR, Ivanac G, Đudarić L, Vujević F, Brkljačić B. Spontaneous pneumomediastinum, pneumothorax and subcutaneous emphysema: Radiological aspects of rare COVID-19 complications in 3 patients. Radiol Case Rep 2021; 16: 3237–43.
79 Alavian N, Stephens JR, DeWalt DA. Spontaneous Pneumomediastinum in a Patient with COVID-19 Pneumonia. J Gen Intern Med 2021; 36: 2845–6.
80 Bozan Ö, Atiş ŞE, Çekmen B. A rare complication of Covid-19: Spontaneous pneumothorax following pneumomediastinum; case report. Am J Emerg Med 2021; 47: 342.e1-342.e2.
81 Hogan G. COVID-19-Associated Pneumomediastinum: An Emerging Clinical Presentation. Cureus 2021; 13: e18287.
82 Kasturi S, Muthirevula A, Chinthareddy RR, Lingaraju VC. Delayed recurrent spontaneous pneumothorax post-recovery from COVID-19 infection. Indian J Thorac Cardiovasc Surg 2021; 37: 551–3.
83 Iuorio A, Nagar F, Attianese L, et al. Spontaneous Pneumomediastinum and Pneumothorax in Nonintubated COVID-19 Patients: A Multicenter Case Series. Am J Case Rep 2021; 22: e933405.
84 S. Rashid Ali MR. The first reported use of autologous blood pleurodesis for treatment of prolonged air leak in COVID-19-related spontaneous pneumomediastinum and pneumothorax: A case report. Respirol Case Rep 2021; 9: e0840.
85 Komiya K, Hamanaka R, Shuto H, et al. Re-expansion pulmonary edema following a pneumothorax drainage in a patient with COVID-19. BMC Pulm Med 2021; 21: 293.

86 Fantin A, Castaldo N, Vailati P, Morana G, Patruno V. Full medical treatment of COVID-19 associated large pneumothorax - A case report. Monaldi Arch Chest Dis 2021; 92. DOI:10.4081/monaldi.2021.1956.
87 Gutierrez-Ariza JC, Rodriguez Yanez T, Martinez-Ávila MC, Almanza Hurtado A, Dueñas-Castell C. Pneumomediastinum and Pneumothorax Following Non-invasive Respiratory Support in Patients With Severe COVID-19 Disease. Cureus 2021; 13: e18796.
88 Shah S, Pokhrel A, Chamlagain R, et al. Case report of a spontaneous pneumothorax after the recovery from COVID-19 pneumonia: A delayed complication. Clin Case Rep 2021; 9: e04971.
89 Shah S, Pokhrel A, Chamlagain R, et al. Case report of a spontaneous pneumothorax after the recovery from COVID-19 pneumonia: A delayed complication. Clin Case Rep 2021; 9: e04971.
90 Mitsuyama Y, Tanaka S, Ike A, Tanaka J, Fujimi S. Refractory pneumothorax secondary to COVID-19 treated by autologous blood patch pleurodesis. QJM 2021; : hcab254.
91 Polistina GE, Lanza M, Di Somma C, Annunziata A, Fiorentino G. A Rare Evolution to Pneumopericardium in Patient with COVID-19 Pneumonia Treated with High Flow Nasal Cannula. Medicina (Kaunas) 2021; 57: 1122.
92 Ulutas H, Celik MR, Gulcek I, et al. Management of spontaneous pneumothorax in patients with COVID-19. Interactive CardioVascular and Thoracic Surgery 2021; : ivab280.
93 Habib MB, Mohammad Obeidat I, Ali K, Abdelrazek M, Mohamed MFH. Bronchopleural fistula causing persistent pneumothorax in COVID-19 pneumonia patient with no risk factors. Clin Case Rep 2021; 9: e05128.
94 Panico R, Cai J, Butts CA, To JQ. Understanding the course of COVID-19-induced pneumomediastinum. JAAPA 2021; 34: 31–3.
95 Ufuk F, Yavas HG, Kis A. An unusual cause of spontaneous pneumothorax: Post-COVID-19 pulmonary fibrosis. Am J Emerg Med 2021; 49: 440.e5-440.e6.
96 Saha BK, Bonnier A, Chong WH, Chenna P. Successful use of endobronchial valve for persistent air leak in a patient with COVID-19 and bullous emphysema. BMJ Case Rep 2021; 14: e246671.
97 Abbas M, Alibrahim H, Hasan M, Khouri A, Sawaf B, Swed S. Subcutaneous emphysema and spontaneous pneumomediastinum in non-intubated COVID-19 patient: The first case report in Syria. Ann Med Surg (Lond) 2021; 72: 103074.
98 Al Armashi AR, Somoza-Cano FJ, Patell K, et al. Spontaneous pneumomediastinum: A collaborative sequelae between COVID-19 and self-inflicted lung injury - A case report and literature review. Radiol Case Rep 2021; 16: 3655–8.
99 Endres F, Spiro JE, Bolt TA, et al. One-year follow-up—case report of secondary tension pneumothorax in a COVID-19 pneumonia patient. Infection 2022; 50: 525–9.
100 de Albuquerque JHC, da Silva AMHP, de Almeida TÍF, Farias LABG. COVID-19 and spontaneous pneumomediastinum: a rare complication. Rev Soc Bras Med Trop; 54: e0871-2020.
101 Mallick T, Dinesh A, Engdahl R, Sabado M. COVID-19 Complicated by Spontaneous Pneumothorax. Cureus; 12: e9104.
102 Natarajan P, Skidmore J, Aduroja O, Kunam V, Schuller D. Bilateral pneumatoceles resulting in spontaneous bilateral pneumothoraces and secondary infection in a previously healthy man with COVID-19. Proc (Bayl Univ Med Cent); 34: 590–2.
103 Sahagun J, Chopra A, David AG, Dao D, Chittivelu S. Secondary Spontaneous Pneumothorax in a COVID-19 Recovered Patient. Cureus; 13: e16415.
104 Younes I, Mohammadian M, Elkattawy S, Singh Z, Brescia ML. SARS-CoV-2 Associated With Pneumothorax: A Case Report and Literature Review. Cureus; 12: e12191.

Supplementary Table S4. Clinical characteristic and outcome of patients according to presenting clinical scenarios

Factor	Group A (Initial presentation)	Group B (During hospitalization)	Group C (Recent recovery of COVID-19)	*p-value*
n	n=68	n=65	n=18	
Age	55.0 [37.0, 67.0]	60.0 [45.0, 70.0]	53.5 [40.0, 63.5]	*0.134*
Gender				*0.083*
Female	14 (20.9)	9 (13.8)	0 (0.0)	
Male	53 (79.1)	56 (86.2)	18 (100.0)	
**Comorbidities**				
With Comorbidity	36 (56.2)	37 (57.8)	7 (38.9)	*0.345*
Hypertension	14 (22.2)	23 (37.1)	2 (11.8)	*0.053*
Obesity	11 (17.5)	5 (8.1)	1 (5.9)	*0.192*
Respiratory diseases	9 (14.3)	3 (4.8)	4 (23.5)	*0.058*
**COVID-19 targeted therapy**				
Steroids	22 (48.9)	40 (66.7)	6 (60.0)	*0.186*
Remdesivir	4 (11.8)	14 (28.6)	1 (11.1)	*0.134*
Convalescent plasma	0 (0.0)	5 (10.9)	2 (25.0)	*0.039*
Intravenous immunoglobulin	0 (0.0)	4 (8.3)	0 (0.0)	*0.160*
Lopinavir/ritonavir	5 (14.7)	4 (8.3)	1 (12.5)	*0.659*
Tocilizumab	3 (8.8)	8 (16.7)	2 (25.0)	*0.410*
**Presenting symptoms**				
Chest pain	25 (47.2)	16 (31.4)	14 (77.8)	*0.003*
Cough	32 (50.0)	9 (17.6)	4 (22.2)	*0.001*
Fatigue	5 (9.4)	1 (2.0)	0 (0.0)	*0.123*
Fever	38 (57.6)	11 (19.3)	1 (5.6)	*<0.001*
Dyspnea	55 (85.9)	43 (81.1)	15 (83.3)	*0.782*
Oxygen saturation at room-air	85.5 [79.8, 91.0]	82.0 [78.5, 84.8]	88.0 [84.0, 95.0]	*0.107*
**PNx/PMEx characteristics**				
Subcutaneous emphysema	18 (27.3)	20 (33.9)	2 (11.8)	*0.197*
Tension PNx	7 (12.7)	6 (10.0)	5 (29.4)	*0.116*
Type.of.diseases (%)				*0.093*
PMEx only	24 (35.8)	17 (26.2)	2 (11.1)	
PNx only	28 (41.8)	33 (50.8)	14 (77.8)	
PNx with PMEx	15 (22.4)	15 (23.1)	2 (11.1)	
Lesion_PNx (%)				*0.709*
Bilateral	9 (23.7)	6 (12.8)	2 (12.5)	
Left	13 (34.2)	19 (40.4)	7 (43.8)	
Right	16 (42.1)	22 (46.8)	7 (43.8)	
**Radiologic findings of chest CT**				

Emphysema	4 (7.1)	7 (11.1)	0 (0.0)	*0.311*
Ground glass opacities	41 (75.9)	50 (86.2)	14 (82.4)	*0.375*
Pleural effusion	7 (12.7)	2 (3.2)	2 (11.8)	*0.141*
Visible bullae	5 (9.1)	6 (9.5)	5 (29.4)	*0.057*
**Managements**				
Chest tube insertion	33 (61.1)	33 (56.9)	15 (83.3)	*0.126*
Conservative care¶	31 (57.4)	37 (59.7)	4 (22.2)	*0.015*
Surgery	6 (10.7)	2 (3.1)	0 (0.0)	*0.109*
Non-invasive ventilation	4 (7.4)	5 (8.2)	0 (0.0)	*0.463*
Invasive mechanical ventilation	17 (26.2)	11 (18.0)	2 (11.1)	*0.296*
**Outcome**				
Hospital stay, days	10.0 [4.0, 18.0]	15.0 [11.0, 26.0]	7.0 [3.0, 13.8]	*0.003*
ICU admission	23 (41.1)	25 (39.7)	3 (16.7)	*0.152*
Mortality	20 (29.9)	14 (21.5)	1 (5.6)	*0.087*

All data were presented as n(%), n/N(%), or median [interquartile range(IQR)]

CT, Computes tomography; ICU, intensive care unit; PMEx, pneumomediastinum; PNx, pneumothorax

¶ It means close monitoring and/or supplemental oxygen support through nasal cannula and reservoir mask.

Supplementary Table S5. The Causes of death among deceased patients (n=35)

Causes of Death	Non-survivors<br>n=35 (23.2 % of total patients)
Respiratory failure	15 (42.9%)
Cardiovascular disease	1 (2.9%)
Sepsis	4 (11.4%)
Multi-organ failure	4 (11.4%)
Unknown	11 (31.4%)

Supplementary Figure S1. PRISMA flow diagram of selection processes

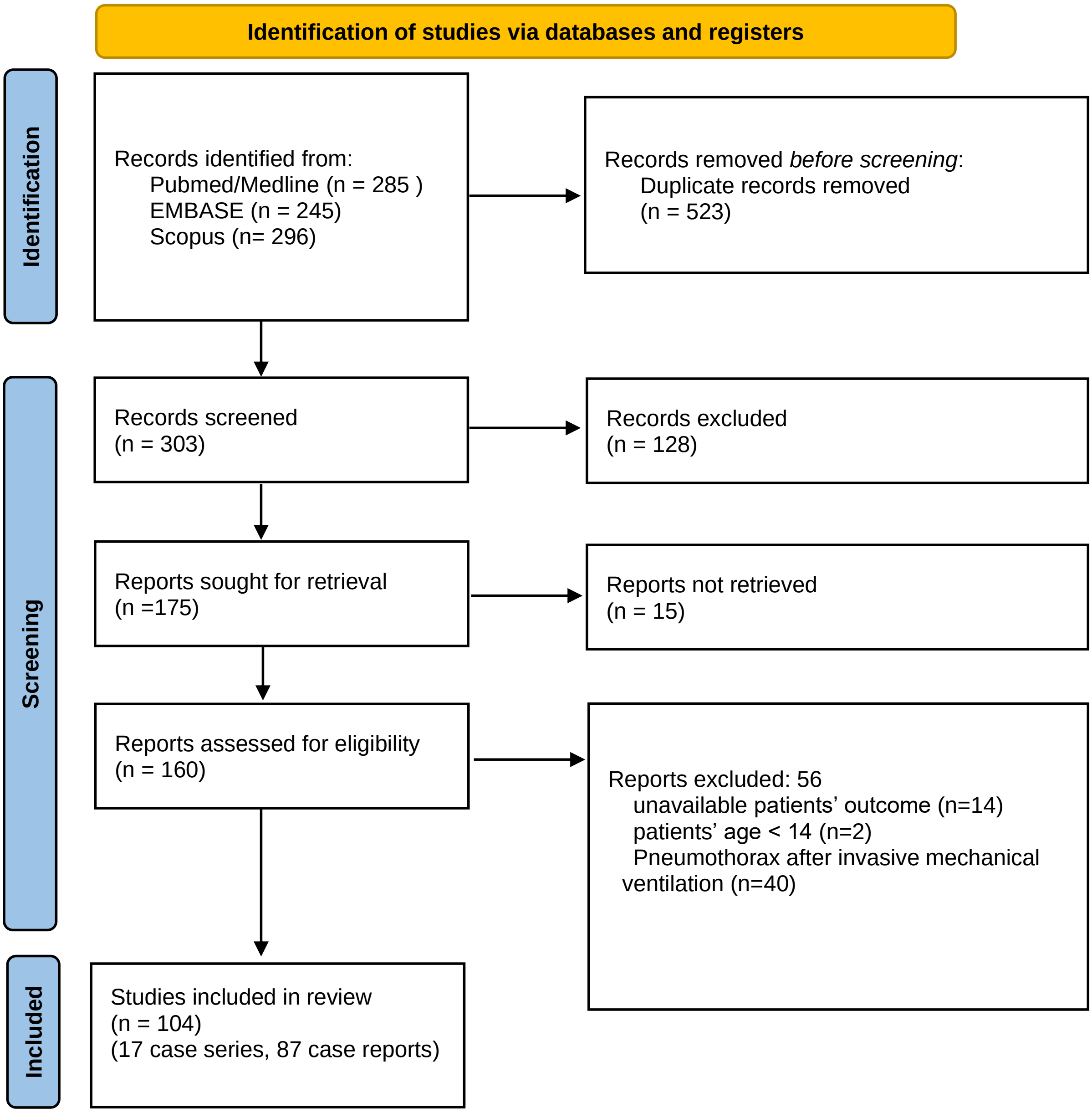

Supplement: Supplementary file 1 [file jcm-11-07132-s001.zip › jcm-2014205-supplementary.pdf]
